# Clinical Study and Profile of Ocular Trauma: Findings From a Rural Hospital in Central India

**DOI:** 10.7759/cureus.26915

**Published:** 2022-07-16

**Authors:** Vishal Wagh, Pravin Tidake

**Affiliations:** 1 Ophthalmology, Jawarhalal Nehru Medical College, Datta Meghe Institute of Medical Sciences, Wardha, IND

**Keywords:** hyphema, road traffic accident, blunt ocular trauma, closed globe injury, open globe injury

## Abstract

Objective

In this study, we aimed to examine the presence of visual damage due to ocular trauma and assess visual outcomes, and document the clinical spectrum and outcomes following ocular injuries among patients presenting to a tertiary care hospital in rural central India.

Methods

This was a hospital-based prospective interventional study conducted over a period of two years from August 2019 to August 2021. Patients with ocular injuries attending the casualty and the Ophthalmology department were included in the study after applying the inclusion and exclusion criteria. A detailed and comprehensive ophthalmic examination was performed and visual acuity was noted at the presentation and follow-ups. The patients were followed up at regular intervals, initially at one week and subsequently at three and six weeks.

Results

The incidence of ocular trauma was highest in the age group of 31-40 years. There were only three patients aged more than 60 years; 15 were in the pediatric age group (1-20 years). The ocular trauma was highest in males (88.33%) than in females (11.67%). The majority of the patients were from rural areas (70%) and 30% were from urban areas. In this study, both eyes were equally involved. The right eye was involved in 45% of patients and the left eye was involved in 55%. Of note, 80% of the patients presented with closed globe injury, and 20% presented with open globe injury. On classifying the open and closed globe injuries into their subtypes, it was observed that the majority of the patients had lid laceration (n=43, 71.67%), followed by corneal penetration in 12 patients (20%), corneal abrasion in three patients (5%), and lid abrasion in two patients (3.33%). While 34 patients presented to the hospital with a history of road traffic accidents (56.67%), accidental trauma (by a wooden stick, hook of a blouse, bangle, etc.) was noted in 17 patients (28.33%), trauma by soil particle and hand pump in five patients (8.33%), and electrical trauma caused by the blast of capacitor in one patient (1.67%). One patient (1.67%) had sustained trauma from a piece of wood while working on the farm and two patients (3.33%) had a trauma because of assault. Thirty-four patients (66.67%) had a history of falls from bikes, and the next most common object causing trauma was a wooden piece/stick (four patients, 6.67%), followed by trauma caused by an iron particle in four patients (6.67%), trauma by stone in three patients (5%), and trauma by hand pump in two patients (3.33%).

Conclusion

Road traffic accidents were the most common cause of ocular trauma in patients attending this rural hospital in central India. The ocular structures involved and types of ocular trauma play a significant role in determining the visual outcomes in these patients.

## Introduction

A significant number of blindness cases worldwide are related to ocular trauma. Over two million cases of ocular trauma are reported every year, of which over 40,000 result in significant vision loss. Traumatic ocular injury negatively affects the quality of life of the patients and their families, as well as their socioeconomic status and psychological well-being. Injury to a person or a tissue or organ is defined as the interruption of tissue function due to the transfer of external energy (mechanical, thermal, radiant, nuclear, chemical, or electrical) [1]. The eyeball is generally well protected in our bodies. The lids, eyelashes, and orbital margins protect the eye from direct damage. As for physiological protection, it is protected by blink reflex, head-turning reflex, and lacrimation after the entry of any irritating material.

Trauma can cause injuries to the globe, optic nerve, and adnexa of the eye, and these range from superficial to vision-threatening complications. It is critical that ophthalmologists and non-ophthalmologists use a standardized classification system of terminology and assessment when describing and communicating clinical findings as our understanding of the pathophysiology and management of these disorders has evolved tremendously in recent decades. The ocular tissue is delicate and sensitive, making ocular injuries more severe than those involving other body parts, and may lead to permanent blindness. Hence, to facilitate proper communication between ophthalmologists and plan clinical trials in the field of ocular trauma, the Ocular Trauma Score (OTS) has been developed. There are a variety of ocular injuries, including minor ones, such as a subconjunctival hemorrhage that does not affect vision, and more severe ones such as a globe rupture or retinal detachment. Globe injuries are classified into two types: open and closed. The delicate, sensitive nature of the ocular tissues causes ocular injuries to affect the eye more severely than any other part of the body, often leading to permanent blindness.

Globally, ocular trauma is the most common cause of visual disability and morbidity. In India, over 500 lakh people suffer from blindness, and every year the blind population increases by 38 lakhs. Of note, 1.2% of cases of blindness are caused by avoidable ocular injuries. A rural population (4.5%) may have a higher prevalence of blindness compared to an urban one (3.97%) [2]. People living in rural areas are often uninformed about protective devices such as goggles and shields. Agricultural work and handling of animals are also major causes of eye injuries.

Due to the financial costs associated with ocular trauma and the economic burden on the healthcare system resulting from the cost of treatment and rehabilitation services, raising awareness and implementing prevention measures are highly justified. Many injuries can be prevented by increasing public awareness about potential risk factors and agents that may cause injury [3-4]. The incidence of ocular trauma in India reportedly ranges from 1 to 5% [5]. In light of these, this study aims to provide epidemiological data on ocular injury based on our analysis of patients at a hospital in rural central Maharashtra. We believe that this data can contribute to efforts toward planning and providing improved eye care as well as implementing preventive measures.

## Materials and methods

Study design and enrollment

The ethical approval to perform the study was obtained from the Datta Meghe Institute Medical Ethical Committee (DMIMS(DU)/IEC/SEPT-2019/8363). This was a hospital-based observational study conducted at a rural hospital in central India spanning a period of two years from August 2019 to August 2021. Patients with ocular injuries attending the casualty and the Ophthalmology department were selected for the study based on the inclusion and exclusion criteria.

Inclusion criteria

Patients with ocular injuries reporting to the casualty and the ophthalmology OPD who were aged between 1-80 years were included in the study.

Exclusion criteria

Patients aged less than one year and those aged more than 80 years, those carrying war-related injuries, thermal injuries, ultrasonic injuries, radiation injuries, chemical injuries, orbital injuries with fractures, and patients who failed to attend regular follow-ups were excluded.

Sample size

A total of 60 patients with ocular trauma who presented to the hospital during the study period were enrolled (based on a 95% confidence interval, the prevalence of ocular trauma in rural population of central India = 2.4% [6], and a desired margin of error = 4% = 0.04).

Examination

The patients who required admission were admitted and managed after proper examination. At the initial examination, Snellen charts were used to assess the patients' vision. Both direct and indirect pupillary reaction was checked. A thorough and careful examination of the fundus was done using a direct and indirect ophthalmoscope. Intraocular pressure (IOP) was measured using Schiotz or Goldmann applanation tonometer. Then the patients were managed after obtaining informed consent. The patients were followed up at regular intervals, initially at one week and subsequently at three and six weeks. At every visit, the patients underwent a detailed ocular examination, which included a vision assessment using the Snellen chart and a slit-lamp examination. Changes, if any, were noted at each visit.

## Results

This study included patients with ages ranging from one to >60 years. Most of the patients were in the age group of 31-40 years, i.e., in the fourth decade. It was found that elderly patients (aged >60 years) had the least number of ocular traumas (Table 1).

**Table 1:** Distribution of patients according to their age SD: standard deviation

Age group (years)	Number of patients	Percentage
1-10	7	11.67
11-20	6	10
21-30	15	25
31-40	17	28.33
41-50	7	11.67
51-60	5	8.33
>60	3	5
Total	60	100
Mean ± SD: 32.28 ± 16.71 (1-84 years)		

As for gender distribution, out of the total 60 patients, 53 were male and seven were female. Male patients constituted 88.33% and females comprised 11.67% of the total patient population, and the male-to-female ratio was approximately 8:1 (Table 2).

**Table 2:** Distribution of patients according to their gender

Gender	Number of patients	Percentage
Male	53	88.33
Female	7	11.67
Total	60	100

With regard to the residential areas of patients, 70% (n=42) hailed from rural areas and 30% (n=18) resided in urban areas (Table 3).

**Table 3:** Distribution of patients according to the area of residence

Area of residence	Number of patients	Percentage
Rural	42	70
Urban	18	30
Total	60	100

Among the 60 patients, 45% (n=27) had the right eye involved while 55% (n=33) had the left eye involved (Table 4).

**Table 4:** Distribution of patients according to eye involvement

Eye involvement	Number of patients	Percentage
Right eye	27	45
Left eye	33	55
Total	60	100

Of note, 20% (n=12) of patients presented with an open globe injury, and 80% (n=48) presented with a closed globe injury (Table 5).

**Table 5:** Distribution of patients according to the type of injury

Type of injury	Number of patients	Percentage
Open globe	12	20
Closed globe	48	80
Total	60	100

**Figure 1:**
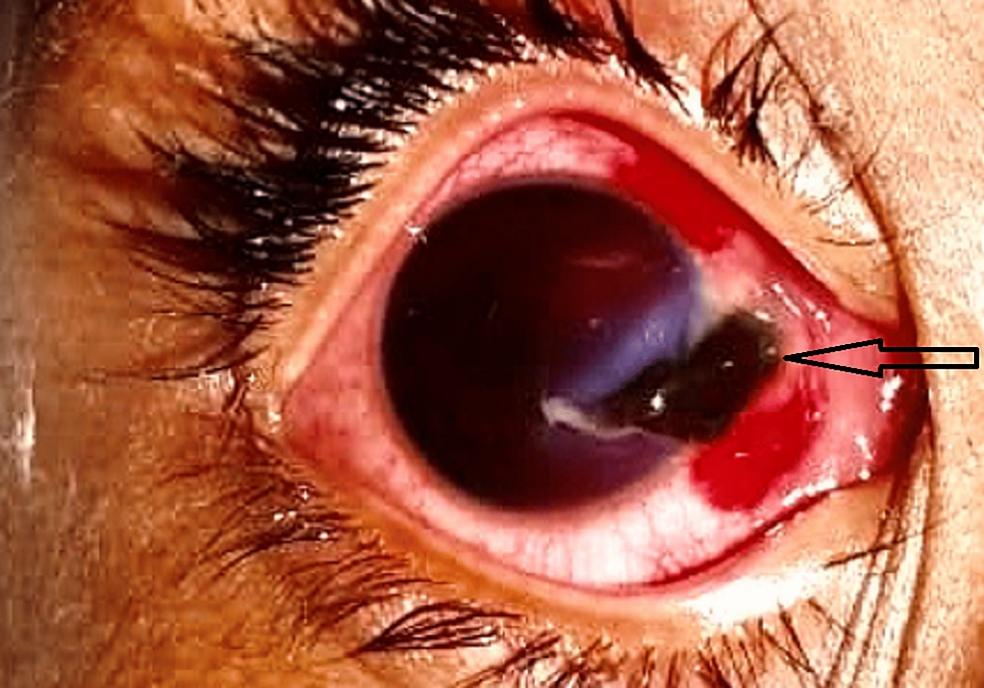
Corneal tear with iris prolapse (arrow)

**Figure 2:**
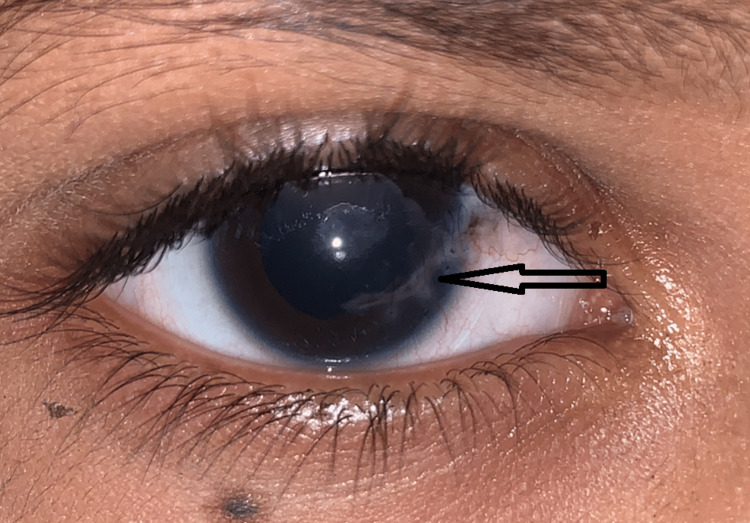
Sutured corneal tear with the correction of iris prolapse (arrow)

In our study, on classifying the open globe and closed globe injuries into subtypes of ocular trauma, it was observed that a majority of patients had lid laceration (71.67%), followed by corneal penetration in 20% (Figures 1, 2) and further by corneal abrasion and lid abrasion in 5% and 3.33% respectively (Table 6).

**Table 6:** Distribution of patients according to injury subtypes

Injury subtype	Number of patients	Percentage
Corneal abrasion	3	5
Corneal penetration	12	20
Lid abrasion	2	3.33
Lid laceration	43	71.67
Total	60	100

It was noted that 56.67% of patients (n=34) had a history of falls while 28.33% (n=17) had a history of some accidental trauma (e.g., by glass particles); 1.67% (n=1) had a history of trauma related to an electrical cause (due to blast of capacitor), and two patients (3.34%) had a history of experiencing an assault (Table 7).

**Table 7:** Distribution of patients according to the mode of injury

Mode of injury	Number of patients	Percentage
Road traffic accident	34	56.67
Accidental	17	28.33
Trauma	5	8.33
Electrical	1	1.67
Agriculture-related trauma	1	1.67
Assault	2	3.33
Total	60	100

As shown in Table 8, the majority of patients reported to the hospital with a history of falls (66.67%; due to road traffic accidents or falls at home) followed by trauma due to wooden stick/piece and iron particles (6.67% each), and 5% of patients had a history of trauma caused by stone or stone particles. Two patients each sustained trauma caused by glass particles and hand pumps (Table 8).

**Table 8:** Distribution of patients according to the cause of trauma

Cause of trauma	Number of patients	Percentage
Glass particle	2	3.33
Blast of capacitor	1	1.67
Blunt object	1	1.67
Bull horn	1	1.67
Fall	40	66.67
Hand pump	2	3.33
Blouse hook	1	1.67
Iron particle	4	6.67
Mobile charger	1	1.67
Wooden piece/stick	4	6.67
Stone	3	5.00
Total	60	100

On further classifying the open globe injuries based on the zone of injury, 18.33% had an injury in zone 1, 1.67% had an injury in zone 3, while two patients (3.33%) had an injury in both zones 1 and 2 (corneoscleral tear) (Table 9).

**Table 9:** Distribution of patients according to the zone of injury

Zone of injury	Number of patients	Percentage
Zone 1	11	18.33
Zone 2	0	0
Zone 3	1	1.67
Zone 1 + 2	2	3.33
Total	13 (out of 60)	21.67

Table 10 depicts the visual acuity of ocular trauma patients at presentation, after one week, at three weeks, and at six weeks. It shows a significant increase in visual acuity in patients after they have undergone a proper follow-up (Table 10).

**Table 10:** Distribution of patients according to visual acuity at different time points NS: not significant

Visual acuity	At presentation	1 week	3 weeks	6 weeks
6/6	14 (23.33%)	20 (33.33%)	24 (40%)	27 (45%)
6/9	13 (21.67%)	17 (28.33%)	15 (25%)	13 (21.67%)
6/12	14 (23.333%)	7 (11.67%)	5 (8.33%)	7 (11.67%)
6/18	3 (5%)	1 (1.67%)	2 (3.33%)	1 (1.67%)
6/24	1 (1.67%)	1 (1.67%)	4 (6.67%)	3 (5%)
6/36	1 (1.67%)	4 (6.67%)	2 (3.33%)	2 (3.33%)
6/60	0 (0%)	1 (1.67%)	1 (1.67%)	1 (1.67%)
Counting fingers	5 (8.33%)	4 (6.67%)	2 (3.33%)	2 (3.33%)
Perception of light present/projection of rays accurate	5 (8.33%)	1 (1.67%)	2 (3.33%)	1 (1.67%)
Perception of light present/projection of rays inaccurate	2 (3.33%)	2 (3.33%)	1 (1.67%)	1 (1.67%)
No perception of light	2 (3.33%)	2 (3.33%)	2 (3.33%)	2 (3.33%)
Total	60 (100%)	60 (100%)	60 (100%)	60 (100%)
Χ^2^ value		10.50	13.28	14.07
P-value		0.39, NS	0.20, NS	0.16, NS

Since the majority of patients presented with lid laceration, lid suturing was the main intervention performed in our study. One patient with a corneoscleral tear had a painful blind eye and hence evisceration was performed on that patient (Table 11).

**Table 11:** Distribution of patients according to interventions performed

Intervention	Number of patients	Percentage
Lid suturing	43	71.67
Foreign-body removal	4	6.67
Corneal tear repair	6	10.00
Medical management	3	5.00
Corneal tear repair + iris cyst excision	1	1.67
Corneal tear repair + cataract extraction + optical iridectomy	1	1.67
Evisceration	1	1.67
Corneal perforation sealed with cyanoacrylate glue with cataract extraction	1	1.67
Total	60	100

## Discussion

This study involved 60 patients with ocular trauma who presented to a tertiary eye care center in rural central India. After performing proper interventions, we observed that 79.34% of them achieved a final visual acuity of 6/18 or better, fulfilling the WHO criteria for no or mild visual impairment [7].

In our study, the majority of ocular trauma patients (28.33%) were in the age group of 31-40 years. The mean age of the patients was 32.28 ± 16.71 years. Poy Raiturcar et al. [8] conducted a study among 500 patients, and they reported that the prevalence of ocular injuries was highest in the age group of 21-40 years (45%). Kumar and Vishwas [9], in their study of 60 patients, found middle-aged males (36-55 years) to be the age group with the highest incidence (43.33%). In the study by Singh et al. [10] on pediatric ocular trauma in central India, the incidence was 12.8%.

In our study, males constituted 88.33% of the patient population while females comprised 11.67%, resulting in a male-to-female ratio of 8:1. A study done by Agrawal et al. [11] had a cohort with males comprising 84.8% of the total and females making up 15.2%. A study by Karve et al. [12] found that males were affected 3.7 times more than females.

Our study found that out of the total 60 patients, 45% (n=27) had the right eye involved, while 55% (n=33) had the left eye involved. Almost all injuries were unilateral. Misra et al.'s [4] study has shown that most ocular injuries are unilateral. Additionally, both eyes were injured almost in equal numbers, with 49% of injuries affecting the right eye and 49.25% affecting the left eye. A study by Maiya et al. [5] observed the right eye and left eye involvement to be equal, with the right eye accounting for 50.52% of injuries and the left eye constituting 49.48%.

The most common etiology encountered was falls due to road traffic accidents (n=34, 56.67%). It was followed by some type of accidental trauma. Agriculture-related trauma was the least common cause (1.67%) in our study. The most common mode of injury was road traffic accidents (56.67), which is in line with the 40% rate found in an ocular trauma study done in Karnataka by Kumar et al. [9]. Another study by Karve et al. [11] found that most ocular traumas occurred due to blunt objects (25.75% of cases). Similar results were found in a study by Nirmalan et al. [13], which reported that blunt objects were the most common cause of injury in their study (54.9%).

Our study had 48 patients (80%) with closed globe injuries and 12 with open globe injuries (20%). In a study by Shukla et al. [14], 66.7% had closed globe injuries, whereas 26.7% had open globe injuries. Poy Raiturcar et al.'s [8] study showed that closed globe injuries were seen in 450 (90%) patients, while 26 (5.2%) had open globe injuries.

In our study, adnexal injury accounted for 71.67% of the total. Some of the cases showed subconjunctival hemorrhage. Corneal penetration was found in 20% of patients. In a study by Laishram et al. [15], 46.15% of cases had adnexal injuries, followed by 31.92% with contusion injuries, and a globe rupture was the least common type of injury. Muralidhar et al. [16] studied 40 patients with ocular trauma due to road traffic accidents. The most common type of injury in their study was subconjunctival hemorrhage, constituting 70% of cases (28/40), followed by ecchymosis, constituting 50% (20/40).

Our study found that patients presenting early after receiving a blunt ocular trauma and having pathologies such as a black eye, subconjunctival hemorrhage, corneal abrasions, and corneal edema regained normal vision after proper management and timely intervention; 13 patients (21.66%) in our study regained near-normal (visual acuity of 6/9) and 27 patients (45%) regained normal vision (visual acuity of 6/6).

A study done by Pai et al. [17] found that 18 of 32 patients (56.25%) had a best-corrected visual acuity of 6/9 or better at presentation. Of the seven patients (21.87%) having corneal epithelial defects, three patients (9.37%) had a visual acuity of less than 6/9, which improved after the healing of the epithelial defect. After conservative management for hyphema, the condition of three patients (9.37%) improved to best-corrected visual acuity of 6/18 or better with a resolution of hyphaema. 

Our study had more patients from rural areas compared to urban areas as the study was conducted at a rural hospital. Patients from urban areas constituted only 30% of our study population. Most of the patients (34, 56.67%) presented with a history of road traffic accidents to this rural hospital as the road connectivity is good near this hospital. Seventeen patients (28.33%) had sustained some kind of accidental trauma to the eye, e.g., trauma caused by a mobile charger, blouse hook, or trauma caused by bangles. Two patients (3.33%) presented with a history of assault.

Table 12 provides a comparison of our study with other studies on anterior segment pathologies.

**Table 12:** Comparison of our study with other studies on anterior segment pathologies

Ocular involvement	Our study	Pai et al. [16]	Zagelbaum et al. [18]
Lid laceration	71.67%	31.2%	13%
Corneal abrasion	5%	21.8%	23%
Hyphema	6.67%	12.5%	5%
Iris injury	10%	15.5%	4%
Subconjunctival hemorrhage	16.67%	37.5%	23%
Traumatic cataract	5%	-	2%

In our study, one patient had a corneal penetrating injury with traumatic mature cataract with iris incarceration. IOP was normal digitally, and the corneal penetration was sealed with cyanoacrylate glue after removing the traumatic cataract, and the intraocular lens placement was done. One patient had a penetrating injury sustained while engaged in welding work. This patient presented with conjunctival congestion, and hence evisceration was done using the flower petal technique with implant placement with conformer. After four weeks, the patient underwent prosthetic eye placement.

In our study, one patient had trauma caused by a blunt object during an assault incident. The patient had no perception of light at presentation and had a corneoscleral tear. After explaining the prognosis, the patient was operated on under nil visual prognosis, and on follow-up, the patient was found to have developed phthisis bulbi.

This study has some limitations, primarily relating to its small sample size and the short follow-up period. Further studies with larger sample sizes and more extended follow-up periods are required to observe the long-term outcomes of ocular traumas and analyze the delayed complications.

## Conclusions

Based on our findings, ocular trauma is a cause for concern irrespective of the geographical area, economic status, gender, and occupation of the patients as it causes visual disability that makes a person physically, economically, and psychologically disabled. Agriculture is the major occupation in rural areas in central India, and men in the age group of 31-40 years were found to be predominantly affected in our study as most of the males in this age group are engaged in manual labor to earn a living, which makes them vulnerable to injuries of all sorts. And in this area, males are the most common earning members in families. It is necessary to educate the working class about exercising caution while working as well as gaining awareness about traffic rules to reduce the incidences of road traffic accidents. It is also important to raise awareness about getting treatment immediately following injuries.
